# Grey Zone Lymphomas: Lymphomas with Intermediate Features

**DOI:** 10.1155/2012/460801

**Published:** 2012-04-03

**Authors:** Sylvia Hoeller, Christiane Copie-Bergman

**Affiliations:** ^1^Institute of Pathology, University Hospital Basel, 4031 Basel, Switzerland; ^2^AP-HP, Groupe Henri Mondor-Albert Chenevier, Département de Pathologie, Hôpital Henri Mondor, 51 Avenue du Maréchal de Lattre de Tassigny, 94010 Créteil, France; ^3^Université Paris-Est, Faculté de Médecine, UMR-S 955, 94010 Créteil, France; ^4^INSERM, Unité U955, 94010 Créteil, France

## Abstract

The current classification of lymphoid neoplasms is based on clinical information, morphology, immunophenotype, and molecular genetic characteristics. Despite technical and scientific progress, some aggressive B-cell lymphomas with features overlapping between two different types of lymphomas remain difficult to classify. The updated 2008 World Health Organization (WHO) classification of Tumours of the Hematopoietic and Lymphoid Tissues has addressed this problem by creation of two new provisional categories of B-cell lymphomas, unclassifiable; one with features intermediate between diffuse large B-cell lymphoma and classical Hodgkin lymphoma and the second with features intermediate between diffuse large B-cell lymphoma and Burkitt lymphoma. We review here the diagnostic criteria of these two provisional entities and discuss new scientific findings in light of the 2008 WHO classification.

## 1. Introduction

The current classification of lymphoid neoplasms is based on clinical information morphology, immunophenotype, and molecular genetic characteristics. Most lymphomas can be accurately classified. However, some lymphomas present with features transitional between diffuse large B-cell lymphomas (DLBCLs) and classical Hodgkin lymphoma (cHL) or DLBCL and Burkitt lymphoma (BL), and these are difficult to classify [1]. These lymphomas have been reported in the literature using different terms, such as borderline lymphomas, B-cell lymphomas unclassifiable, atypical Burkitt lymphoma, Burkitt-like lymphomas, or gray zone lymphomas. The term “Gray Zone Lymphoma” was firstly used in 1998 at the “Workshop on Hodgkin's disease and related diseases” to designate lymphomas at the border of cHL and other entities [2]. This term was then further extended to lymphomas with overlapping features between BL and DLBCL. The 2008 updated WHO classification of Tumours of the Hematopoietic and Lymphoid Tissues proposed to assign these gray zone lymphomas to provisional categories called B-cell lymphomas unclassifiable with features intermediate between DLBCL and cHL (BCLu-DLBCL/cHL) and B-cell lymphomas unclassifiable with features intermediate between DLBCL and BL (BCLu-DLBCL/BL) [3]. The reason to create these provisional categories is to enable to collect for further studies and to maintain the “purity” of well-defined categories. This would be particularly relevant for conducting clinical studies. This paper focuses on these two provisional entities introduced in the 2008 WHO classification of Tumours of the Hematopoietic and Lymphoid Tissues.

## 2. B-Cell Lymphoma, Unclassifiable, with Features Intermediate between Diffuse Large B-Cell Lymphoma and Classical Hodgkin Lymphoma (BCLu-DLBCL/cHL)

Primary mediastinal diffuse large B-cell lymphoma (PMBCL) and classical Hodgkin lymphoma of nodular sclerosing subtype (cHL-NS) have clinical, histopathological, and molecular similarities (Table 1). Both lymphomas present as an anterior mediastinal mass with involvement of the thymus and/or supraclavicular lymph nodes and affect preferentially young women. Median age of presentation is slightly older in PMBCL (35 years) than in cHL-NS (30 years) [4]. The histopathological features of PMBCL include a diffuse proliferation of large cells with clear abundant cytoplasm and fine compartmentalizing sclerosis. Reed-Sternberg-like cells may be present [5, 6], and distinction from cHL-NS can sometimes be difficult. The neoplastic cells in PMBCL express B-cell markers (CD20, CD79a, CD19, PAX5) and lack expression of HLA class I antigens and surface immunoglobulin (Ig). However, expression of Ig-associated transcription factors BOB1, OCT2, and PU1 is preserved in contrast to cHL [7, 8]. CD30 is expressed in 70% of cases and tumour cells are typically CD23 positive. Seventy per cent of PMBCL and 10% of cHL express the MAL protein linking them histogenetically to the thymic asteroid medullary B cells [9, 10]. EBV is absent in PMBCL.

On gene expression profiling studies, the PMBCL gene signature differs from that of germinal centre B-cell-like and activated B-cell-like DLBCL. Highly expressed genes include *MAL*, *Interleukine 4 induced gene 1 (IL4I1*), *TARC*, *NFkB2,* and *PDL1/L2* [11, 12]. Interestingly, the PMBCL gene signature appeared to be more related to cHL gene signature as both represent downregulation BCR pathway signalling, constitutive NF-kappa B activation, activation of the cytokine-JAK-STAT pathway, high expression of extracellular matrix elements, overexpression of the TNF family members, and aberrant activation of the P13K/AKT pathway [11–17]. Recent studies have highlighted many genetic similarities as well. Both entities show gains at 2p15 (*REL* locus) and 9p24 (*JAK2* locus) and breaks at *CIITA* (38% of PMBCL and 15% of cHL) [18–20]. The presence of *CIITA* rearrangement significantly correlates with shorter disease-specific survival for patients with PMBCL [20]. Altogether, these features point to a similar histogenesis.

In recent years, cases with morphological and immunophenotypic features transitional between PMBCL and the nodular sclerosis subtype of cHL have been reported. These cases, which were initially referred to as “gray zone lymphomas,” were assigned in the 2008 WHO classification to a provisional category designated B-cell lymphoma, unclassifiable, with features intermediate between DLBCL and cHL (BCLu-DLBCL/cHL). These lymphomas with intermediate features have been reported mostly from Western countries, and they seem to be less frequent in sub-Saharan Africa and Asia [3]. BCLu-DLBCL/cHL usually presents with mediastinal manifestations, but also include occasional cases involving nonmediastinal lymph node sites. Involvement of lung (by direct extension), liver, spleen, and bone marrow are documented. In contrast to PMBCL, nonlymphoid organs are rarely infiltrated [3]. Interestingly, these lymphomas are probably more frequent in young men and have a more aggressive clinical course and poorer outcome than either cHL or PMBCL [21, 22]. From the morphological point of view BCLu-DLBCL/cHL shows typically sheet-like, confluent growth of pleomorphic tumour cells embedded in a diffusely fibrotic stroma. The majority of tumour cells classically resemble lacunar cells and Hodgkin cells. However, the tumour shows marked variation of morphological aspects ranging from cHL to DLBCL/PMBCL in the same tumour. There is usually a sparse inflammatory infiltrate present with only scattered eosinophils, lymphocytes, and histiocytes. Typically necrotic areas do not include neutrophilic infiltrates. Immunohistochemically, B-cell program is usually preserved in the tumour cells with expression of the transcription factors PAX5, OCT-2, and BOB.1, but this profile is accompanied by expression of typical “cHL markers” like CD15 and CD30. Surface Ig expression is absent. MAL, a typical marker for PMBCL [10], is expressed in at least a proportion of cases [3]. Diagnostic criteria include, for example, cases morphologically resembling PMBCL but with strong expression of CD15, absence of CD20 or presence of EBV [3]. Cases rich in tumour cells resembling cHL, which are strongly positive for CD20 and/or other B-cell markers, are also included in this category [23].

The existence of composite (cHL and PMBCL at the time of diagnosis) or sequential/metachronous lymphomas (cHL following a diagnosis of PMBCL or vice versa) suggests that some lymphomas in the mediastinum show lineage plasticity with a shift over time toward the one or the other entity [21] which may be due to epigenetic and not genetic mechanisms. Based on this consumption Eberle et al. studied the DNA methylation status which is the best established epigenetic marker so far from 10 mediastinal gray zone lymphomas (MGZLs) compared to 10 cHL-NS, 10 PMBCL, and 10 nodal DLBCL cases [24]. MGZL cases had epigenetic profiles intermediate to cHL and PMBCL but clearly distinct from DLBCL. PMBCL and cHL-NS presented with distinct methylation signatures. cHL-NS showed presence of de novo hypermethylation and absence of de novo hypomethylation within CpG islands and in a fraction of promoters outside CpG islands. These results are in line with other studies suggesting that the development of Reed-Sternberg cells may be due to gene silencing by DNA methylation [25, 26]. In contrast, PMBCL showed both de novo hypermethylation and hypomethylation. Interestingly, *HOXA5* hypomethylation was exclusively found in MGZL, and the biological relevance of this finding remains to be explored.

Eberle and colleagues studied the genetic features of 27 MGZL and 6 mediastinal composite or synchronous/metachronous lymphomas by fluorescence in situ hybridization. They demonstrated amplification in 2p16.1 (*REL/BCL11A* locus) and alterations in 9p24.1 (*JAK2/PD2 *locus) in 33% and 55% of the patients, respectively. In addition, rearrangement of the *CIITA* locus at 16p13.13 and gains of 8p24 (*MYC*) were both observed in 27% of the cases each [27]. These very recent findings underline the plasticity of mediastinal BCLu-DLBCL/cHL not only on morphologic and immunophenotypic but also on molecular grounds.

Identification of BCLu-DLBCL/cHL will hopefully enable to better characterize them and above all to develop an optimal therapeutic approach. The clinical management of these patients is actually a challenge for clinicians as cHL and PMBCL require different therapies. Due to the rarity of the disease and the complexity of diagnostic criteria, the optimal therapy for these patients remains unclear. In a small series of patients with mediastinal BCLu-DLBCL/cHL, Traverse-Glehen et al. suggested that patients might benefit better from therapy designed for aggressive B-cell non-Hodgkin lymphoma than those applied to patients with cHL [21]. However, these results need validation on a larger series of patients.

## 3. B-Cell Lymphoma, Unclassifiable, with Features Intermediate between Diffuse Large B-Cell Lymphoma and Burkitt Lymphoma (BCLu-DLBCL/BL)

In order to understand the concept of this new category of lymphoma, we will briefly review the diagnostic criteria for Burkitt lymphoma (BL) according to the updated WHO classification of lymphoid neoplasms.

BL includes three epidemiologic variants, the endemic (so-called African type with 100% EBV association), the sporadic, and the immunodeficiency-associated (mostly HIV infected patients). Additionally, cases presenting with a leukemic picture (formerly known as L3 ALL) and without significant lymphadenopathy are also included. The atypical/Burkitt-like and the plasmacytoid variants are no longer mentioned [23].

The diagnostic criteria for BL are quite strict. Morphologically, the tumour cells are medium sized, with monotonous cytology presenting with round nuclei with finely clumped and dispersed chromatin and multiple basophilic paracentrally situated nucleoli. The cytoplasm is deeply basophilic and usually contains lipid vacuoles (which may better be seen in imprints). The growth pattern is diffuse, and the tumour cells often seem to grow in a cohesive way. The proliferation fraction is extremely high (>90%) with many mitotic figures accompanied by a high fraction of apoptosis and often a starry sky pattern due to the background occurrence of tingible body macrophages [3]. In contrast with previous classification, increased nuclear irregularity, slight nuclear pleomorphism, and/or more prominent, single nucleoli are allowed if the immunophenotype and the molecular characteristics fit with the diagnosis of BL. These lymphomas previously classified as “Burkitt-like” or “atypical Burkitt” lymphoma are now included in the “Burkitt lymphoma” category, and hence the terms “Burkitt-like” or “atypical Burkitt” lymphoma should not be used any longer. This approach is supported by molecular studies, which have revealed that the cases classified morphologically as “atypical” BL have a molecular signature similar to classical BL [23, 28].

The immunohistophenotype required for the diagnosis of BL is strong CD10 and BCL6 positivity, negativity for BCL2, and a Ki67 index of near 100% (at least 90%) [3]. Weak positivity for BCL2 is accepted, but strong expression for BCL2 and a proliferation fraction below 90% are strong contraindications for a diagnosis of BL [23].

On genetics, most cases show rearrangement of* MYC* at 8q24 to the *IG heavy* chain (14q32) or less frequent to the *kappa* (22q11) or *lambda* (2p12) light chain loci. The breakpoints are different in endemic and sporadic BL. Endemic BLs present with breakpoints occurring within the VJ region of the *IGH* locus, while sporadic BL mainly present with breakpoints occurring within IGH switch regions of the *IGH* locus, which may point to the differing maturation status of the two types [29, 30]. Importantly, up to 10% of BL may lack a *MYC* gene translocation by FISH. To date it is not clear if this is due to a failure of detection or if such lymphomas really exist. However, *MYC*-negative BL in adults is characterised by downregulation of microRNA hsa-mir-34b and recurrent duplications of chromosome 11 [28, 31, 32]. However, these *MYC*-negative BLs have to be completely typical on morphological and immunophenotypic grounds to be classified as BL although this opinion is not shared by all authors [1].

 However, in view of the strict diagnostic criteria, there remain cases, which are not completely typical, either on morphological and/or immunohistochemical/genetic grounds and therefore are difficult to assign to either BL or DLBCL category (classical common and distinguishing features between these two entities are listed in Table 2). Gene expression profiling (GEP) studies reveal that though the GEP signature of BL and DLBCL are distinct, in a proportion of B-cell lymphomas the GEP signature is intermediate between BL and DLBCL [28]. Such cases were diagnosed either as BL or DLBCL on morphologic and immunohistochemical grounds by expert hematopathologists, and possibly inappropriate therapy was rendered for some patients. Based on these observations, the recent WHO classification created a provisional entity, B-cell lymphoma, unclassifiable, with features intermediate between DLBCL and BL (BCLu-DLBCL/BL). The reasons for the creation of this new entity are mostly similar to those of BCLu-DLBCL/cHL. Firstly, it intends to collect cases with intermediate features under the same name, and secondly it segregates “clean” BL and DLBCL, which is extremely helpful for clinical trials. On the other hand, it also creates difficulties to clinicians, as the therapeutic strategies differ greatly in adults between BL and DLBCL, and there has been no consensus, on how to treat patients with BCLu-DLBCL/BL.

BCLu-DLBCL/BL are relatively rare and mainly diagnosed in adults [3]. They represent up to 5% of adult aggressive B-cell lymphomas and usually occur in extranodal sites sometimes associated with leukemic involvement [33]. By definition, BCLu-DLBCL/BL harbour intermediate morphological and immunohistochemical features between BL and DLBCL [3]. They may be medium or large cells, usually with a high proliferation fraction and starry sky pattern, with an atypical immunophenotype (lack of CD10 and/or strong BCL2 expression) that precludes the diagnosis of BL. Most of them are of germinal centre subtype with expression of CD10, BCL6 and lack of MUM1 [33].

Cytogenetic characterization of these BCLu has shown that a proportion of them harbours a complex karyotype with two main genetic events—usually c*MYC* alterations together with *BCL2* and/or *BCL6, *less commonly *CCND1* rearrangements, designated the so-called “double hit” lymphomas (DHL). Some patients may have a previous history of low-grade lymphoma such as follicular lymphoma, CLL, or mantle cell lymphoma, and the acquisition of *cMYC* alteration may represent a secondary genetic event [33].

Importantly, lymphomas with a typical DLBCL morphology that have a *MYC* breakpoint are excluded from the category of BCLu-DLBCL/BL. Up to 15% of DLBCL have *MYC* translocations [23, 34], and they are generally associated with an inferior outcome [35, 36].

The clinical evolution of patients with double-hit lymphomas is dramatic with a median survival of 4.5 months, and they are usually resistant to either conventional CHOP-like regimens or to intensive therapy used to treat BL. However, factors associated with a better survival have been identified, which include non-*IGH MYC* partner, BCL2 protein expression, and rituximab inclusive chemotherapy [37].

Altogether, this category of lymphoma appears heterogeneous and remains difficult to diagnose in day-to-day practice based on morphological and immunohistochemical grounds. Interphase FISH with BCL2, BCL6, and *cMYC* DNA probes provides a useful diagnostic tool to identify these DHL. Adult cases in which BL or DLBCL/BL is a diagnostic consideration should be tested for *MYC*, *BCL2,* and *BCL6* rearrangements, and if *MYC* break is associated with *BCL2 *and/or *BCL6* rearrangements, the case should be classified as DLBCL/BL irrespective of other features [23].

 A very recent paper from the group of Reiner Siebert [38] reviewed the “grey zone” between BL and DLBCL from a genetic perspective. This paper aims to clarify the different definitions of intermediate lymphomas and to propose a subclassification based on genetic aberrations. The “intermediate lymphoma” group from GEP studies and the BCLu-DLBCL/BL from the WHO classification are by far not identical. The intermediate group of GEP is defined of a group of lymphomas not meeting the profiling of either molecular BL or for molecular DLBCL. Therefore, this category represents a wastebasket for all lymphomas, which do not fit into the two molecularly defined entities. On the other hand, the BCLu-DLBCL/BL entity defined by the WHO contains all lymphomas, which does not meet the criteria of either BL or DLBCL on morphologic, immunohistochemical, and classical genetic grounds and represents a heterogeneous group of diseases. However, the following aggressive B-cell lymphomas are excluded from BCLu-DLBCL/BL: cases with typical DLBCL morphology with a very high proliferation index, typical DLBCL with a *MYC* translocation, and typical BL in which a *MYC* rearrangement cannot be demonstrated and those with *IG-MYC* rearrangement as the only abnormality, since they probably correspond to real BL with atypical morphology. Salaverria and Siebert [38] proposed a simple approach based predominantly on age and genetic aberrations to classify these aggressive B-cell lymphomas into biologically meaningful and clinically recognizable subgroups.

 In children, the classification into BCLu-DLBCL/BL has currently no influence on therapy or outcome and either intermediate lymphomas according to both GEP and WHO classification are infrequent in patients under the age of 18 years [3, 39] and do not seem to have an adverse prognosis. Almost all childhood “intermediate lymphoma” present with *IG-MYC* fusion, and *BCL2 *breaks are almost always absent. Therefore, it seems that in children “intermediate lymphomas” represent rather true biologic BL, which were classified as “intermediate” in the GEP due to secondary aberrations [38]. In contrast some morphological DLBCLs in children show a GEP signature of molecular BL with more than half of them being *IG-MYC* positive, suggesting that the presence of the *MYC* fusion is mostly responsible for its given molecular signature. Since the *MYC* fusion is very likely to be the first event in lymphoma development, complex karyotypes are indicators of disease progression and inferior outcome and do not indicate an *IG-MYC* fusion as a secondary event in children.

In adult patients the situation is rather different and the subclassification of aggressive B-cell lymphomas has a true impact on treatment decisions and prognosis. Salaverria and Siebert [38] suggest that adult aggressive B-cell lymphomas lacking typical BL morphology and phenotype can be classified thereafter into four different genetic subcategories according to their *MYC* status as follows.

(1) *IG-MYC-Positive Mature Aggressive Lymphomas with Simple Karyotype Lacking Typical BL Morphology and/or Phenotype*. These cases represent a spectrum ranging from cases that would be classified as BL to up to DLBCL based on the WHO classification, since the WHO considers morphology, immunophenotype, and genetic characteristics as being equally relevant [3]. Since the molecular BL signature can also be found in classical DLBCL cases [28], this points to the fact that such cases might be candidates for this molecular BL group.

(2)* IG-MYC-Positive Mature Aggressive B-Cell Lymphomas with Complex Karyotype, Lacking Typical BL Morphology, and/or Phenotype Carrying a High Genetic Complexy*. These cases can correspond to BL with progression or DLBCL with secondary *MYC* break. However, like in the first group it is not easy to set a cutoff between complex and simple karyotype, since there a standard reference method is not defined.

(3) *Non-IG-MYC-Positive Mature Aggressive B-Cell Lymphomas*. *MYC* translocations can involve partners other than the IG heavy or light chain loci. Those translocations are almost exclusively considered as secondary events. These translocations are exceedingly rare in BL but represent up to half of *MYC* translocations in BCLu-DLBCL/BL [3, 28]. Those cases are probably cases with a different primary genetic event (*BCL2*, *BCL6* break, or others) and acquire the *MYC*-break secondarily and may develop very complex karyotypes. 

(4) *Double Hit-Positive Mature Aggressive B-Cell Lymphomas*. These lymphomas carry either *IG-MYC* fusion or non-*IG-MYC* fusion in combination with either *BCL2* and/or *BCL6* breaks. By definition these lymphomas would also conform to the previously defined group. But this specific group comprises of >30% of *MYC*-translocation-positive lymphomas in elderly patients and has an aggressive clinical course. However, Salaverria and Siebert [38] pointed out that further studies are needed to clarify: if double hit lymphomas are different from other *MYC*-positive non-BL cases and if the different types of combinations (DLBCL/BL or DLBCL with *BCL2* and/or *BCL6* break and *IG-MYC* or non-*IG-MYC* fusion) influence the outcome and behavior of these lymphomas.

In addition, there are also *MYC*-translocation-negative aggressive B-cell lymphomas with features intermediate between BL and DLBCL. Here the “intermediate” status is defined according to the WHO criteria by histology and immunophenotype. This group probably represents a heterogeneous group of mainly DLBCL, but little is known about this category to date. Salaverria and Siebert [38] suggest that this genetic classification should be tested for reproducibility and clinical impact in future clinical trials.

In conclusion, since the publication of the 2008 WHO classification, several groups have tried to better characterize these two new provisional entities of B-cell lymphomas, unclassifiable with intermediate features. Eberle and colleagues [24, 27] have shed new insights on the epigenetic and cytogenetic characteristics of BCLu-DLBCL/cHL which highlight the plasticity of the tumour cells and the molecular continuum between PMBCL and cHL. Regarding the second category of B-cell lymphoma, BCLu-DLBCL/BL, the situation is more complex. This category appears extremely heterogeneous and remains difficult to diagnose in day-to-day practice based on morphological and immunohistochemical features. The genetic approach proposed by Salaverria is interesting and supports the fact that the detection of chromosomal abnormalities in the diagnostic workup of aggressive B-cell lymphomas is becoming increasingly important.

Further studies are needed to better define the diagnostic criteria of BCLu-DLBCL/BL and allow clinicians to conduct clinical trials to define the optimal therapy which remains unclear to date.

## Figures and Tables

**Table 1:** Common and distinguishing features of PMBCL, cHL-Nodular sclerosis (NS), and B-cell lymphoma, unclassifiable, with features intermediate between DLBCL and cHL (BCLu-DLBCL/cHL). Modified after Hasserjian et al. [23].

	PMBCL	cHL-NS	BCLu-DLBCL/cHL
Common features			
Age	Young patients	Young patients	Young patients
Gender	Female predominance	Female predominance	Male predominance
Localization	Mediastinal mass eventually supraclavicular lymph nodes	Mediastinal mass eventually supraclavicular lymph nodes	Mediastinal mass eventually supraclavicular lymph nodes or more rarely other lymph nodes
Morphology	Compartmentalizing fibrosis	Fibrosis in thick bands	Confluent, sheet like growth of pleomorphic tumor cells with diffuse fibrotic stroma Variability from area to area
Therapy response	Radiotherapy sensitive	Radiotherapy sensitive	
Immunophenotype	Lack of Ig-Expression Lack of HLA I expression Frequent CD30 expression Expression of MAL and CD23	Lack of Ig-expression Lack of HLA I expression CD30 expression	Transitional features between PMBCL and cHL B-cell program often preserved
Genetic and molcular features	Expression of HLA-I REL (2p15) and JAK2 gains (9p24) *CIITA* breaks Activation: NF-kappaB, JAK-STAT (incl. STAT6), and P13K/AKT pathway High expression of extracellular matrix elements, overexpression of TNF family members	Expression of HLA-I REL (2p15) and JAK2 gains (9p24) *CIITA* breaks Activation: NF-kappaB, JAK-STAT (incl. STAT6), and P13K/AKT pathway High expression of extracellular matrix elements, overexpression of TNF family members	REL (2p15) and JAK2 gains (9p24) *CIITA* breaks

Distinguishing features			
Morphology	Clear cells often homogenous (but Reed Sternberg cells may occur) Little or no inflammatory background	Hodgkin cells and Reed Sternberg cells Typical inflammatory background	
Immunophenotype	B-cell markers preserved (CD20, CD79a, PAX5) B-cell transcription factors present (BOB.1 and OCT-2) CD15 absent Absence of EBV	B-cell markers lacking or only weakly or heterogeneously expressed (especially PAX5) B-cell transcription factors usually negative CD15 may be present EBV may be present	

**Table 2:** Common and distinguishing features of BL, DLBCL, and B-cell lymphoma, unclassifiable, with features intermediate between DLBCL and BL (BCLu-DLBCL/BL).

	BL	DLBCL	BCLu-DLBCL/BL
Common features			
Age	Young children and less frequent young adults	Less frequent in children but frequent in adults of all age groups	Mainly diagnosed in adults
Gender	Male predominance	No real predominance	
Localization	Often extranodal (jaw and iliac region)	Nodal and extranodal	Often extranodal (no predominant region) often widespread disease leukemic presentation is possible
Morphology	Frequent mitotic figures and apoptosis often with starry sky pattern	Frequent mitotic figures and apoptosis may be present	Frequent mitotic figures and apoptosis often with starry sky pattern resembling BL
Immunophenotype	CD10, BCL-6 positive, BCL-2 negative	“BL immunophenotype” (CD10, BCL-6 positive, BCL-2 negative) may be present	Variable depending on morphologic features (see text)
Genetic and molecular features	Typical *IG-MYC* fusion, simple karyotype	Typical *IG-MYC* fusion may be present	Often non-*IG-MYC* fusion complex karyotype

Distinguishing features			
Morphology	Medium-sized blastic cells with basophilic cytoplasm, no inflammatory background, sometimes cohesive growth pattern Small nucleoli at the periphery, mitotic rate always very high (Ki67 >90%)	Pleomorphic large blastic tumor cells, often inflammatory infiltrate, mitotic rate variable	
Genetical and molecular features	Typical *cMYC* fusion with IG light or heavy chain locus, simple karyotype More complex karyotypes possible (sign of progression)	Other types of *cMYC* fusions can be present (other than IG as a partner), complex karyotype possible	Combination of *BCL2* and/or *BCL6* breaks possible (so-called “double or triple hit lymphomas”)
